# Gut mycobiome in the spectrum of Metabolic-dysfunction Associated Steatotic Liver Disease (MASLD): from MASH to hepatocellular carcinoma

**DOI:** 10.1016/j.clinsp.2026.101200

**Published:** 2026-10-16

**Authors:** Cássio Marques Perlin, Melina Belén Keingeski, Bruno de Souza Basso, Beatriz Garcia Mendes, Julliane Dutra Medeiros, Francis Moreira Borges, Bernd Schnabl, Mário Reis Álvares-da-Silva

**Affiliations:** a Graduate Program in Gastroenterology and Hepatology, Universidade Federal do Rio Grande do Sul, Porto Alegre, RS Brazil; b Experimental Laboratory of Hepatology and Gastroenterology, Experimental Research Center, Hospital de Clínicas de Porto Alegre, Porto Alegre, RS, Brazil; c Laboratory of Bacterial Physiology and Microbial Molecular Genetics, Department of Parasitology, Microbiology and Immunology, Universidade Federal de Juiz de Fora, Juiz de Fora, MG, Brazil; d School of Medicine, Department of Internal Medicine, Universidade Federal do Rio Grande do Sul, Division of Gastroenterology, Hospital de Clínicas de Porto Alegre, Porto Alegre, RS, Brazil; e Department of Clinical Analysis, Universidade Federal de Santa Catarina, Florianopolis, SC, Brazil; f San Diego Digestive Diseases Research, Center University of California San Diego, USA

**Keywords:** Gut mycobiome, Metabolic-Dysfunction Associated Steatotic Liver Disease (MASLD), Liver Fibrosis

## Abstract

Gut Microbiome (GM) dysbiosis is associated with the development and progression of Metabolic-dysfunction Associated Steatotic Liver Disease (MASLD) and steatohepatitis (MASH). Human GM contains bacteria, fungi, archaea, and viruses. Gut mycobiome (GMyco) is still poorly studied in patients with MASLD. Herein we investigated GMyco composition at different MASLD stages.

**Methods:**

23 patients were recruited: controls (n = 5); MASH (n = 6); cirrhosis (n = 7); and Hepatocellular Carcinoma (HCC) (n = 5). Stool samples were collected and stored in -80 °C for subsequent DNA extraction and profiled by 18S rRNA sequencing. The single sequence variants obtained were inferred with DADA2 and classified with SILVA v138. Diversity metrics (Simpson; Bray-Curtis PERMANOVA) and DESeq2 with BH FDR were used for statistical analysis.

**Results:**

Among 23 participants, 73.9% were women, and the average BMI was 30.7 kg/m^2^. Current alcohol use was reported in 17.4%, and chronic medication use in 56.5% of individuals. MASH patients showed greater gut mycobiota alpha diversity compared with controls (p < 0.05). Controls exhibited higher relative abundance of the phylum Ascomycota, whereas Basidiomycota predominated in MASH. At the family level, *Aspergillaceae* was more abundant in controls, *Malasseziaceae* and *Sporidiobolaceae* in cirrhosis, and *Saccharomycetaceae* in HCC. Differential abundance analysis identified three ASVs differing between groups, with one ASV (genus Naganishia) homogeneously distributed in MASH and absent in controls.

**Conclusions:**

The gut mycobiota profile varied across the MASLD spectrum, with stage-specific enrichment of certain taxa despite overall similarity in community structure. *Basidiomycetes* ‒ notably *Malassezia* and *Naganishia* ‒ were more abundant in MASH, while *Saccharomyces* dominated in advanced stages. These findings suggest that distinct fungal signatures accompany disease progression and may serve as potential biological markers in MASLD.

## Highlights

• Gut mycobiota plays an important role in liver diseases.
• Little is known about gut mycobiota composition in liver diseases, including MASLD.
• This study suggests that gut mycobiota profile varies with MASLD progression.

**Abbreviations**
AP: Alkaline Phosphatase
ASVs: Single Sequence Variants
AST: Aspartate Aminotransferase
ALT: Alanine Aminotransferase
BMI: Body Mass Index
CN: Healthy Control
CIR: Cirrhosis
DNA: Deoxyribonucleic Acid
DADA2: Divisive Amplicon Denoising Algorithm-2
GM: Gut Microbiome
Gmyco: Gut Mycobiome
GGT: Gamma-Glutamyltransferase
HCC: Hepatocellular Carcinoma
HCPA: Hospital de Clínicas de Porto Alegre
MASLD: Metabolic-dysfunction Associated Steatotic Liver Disease
MASH: Steatohepatitis
NMDS: Non-Metric Multidimensional Scaling.

## Introduction

It has been known for many years that overweight and obesity are associated with fat accumulation in the liver and consequent damage to hepatic cells, leading to chronic inflammation and fibrosis.^1^ This accumulation, which cannot be explained by alcohol intake, is known as Metabolic dysfunction-Associated Steatotic Liver Disease (MASLD).^2^ In its development, factors such as obesity, unhealthy diet, sedentary lifestyle and environmental factors play an important role in its progression.^3^^,^^4^

In the last decade, numerous studies have suggested an association between the Gut Microbiota (GM) and metabolic diseases such as MASLD.^5^^,^^6^ The microbiota consists of bacteria (bacteriota), viruses (virota), fungi (mycobiota) etc., while the individual genomes of bacteria (bacteriome), viruses (virome) and fungi (mycobiome) make up the gut microbiome with 150 times more genes than the human genome itself.^7^^,^^8^ The bacteriome represents 99% of the total microbiome, while the mycobiome is responsible for 0.1% of the remaining genetic load.^9^^,^^10^ The components of the microbiome interact with each other and its composition can be impacted by lifestyle, disease or iatrogenesis through treatment.^11^ Even so, studies on mycobiome are still limited.^12^

The Gut Mycobiome (GMyco) influences the intestinal barrier permeability and the mucus composition, with the high permeability of this barrier resulting in increased liver inflammation, contributing to development and progression of MASLD.^3^^,^^13^ A recent systematic review concluded that individuals with MASLD have a diversified mycobiota than healthy controls.^14^ Fungi may modulate the host immune response and be a risk factor for genetically susceptible individuals.^15^ Fungi contain beta-glucans that hinder the intestinal bile acids reabsorption and increase their synthesis, altering the composition of bile acids and signaling within the intestine-liver axis as well as their effect on hepatic steatosis and cirrhosis.^16^ Thus, this study aims to evaluate the composition of the intestinal mycobiome at different phases of MASLD.

## Material and methods

This study is a single-center cohort that included adults treated at an outpatient clinic of the Gastroenterology Division of Hospital de Clínicas de Porto Alegre (HCPA), Brazil. Only adults with MASLD, diagnosed by the presence of steatosis through ultrasound or liver biopsy, with or without changes in AST and/or ALT, associated with the presence of metabolic syndrome were included in the study. We investigated the history of alcohol use, hemochromatosis, Wilson’s disease, and the history of hepatotoxic drug use. Patients who met the inclusion criteria were enrolled after signing written informed consent. Patients under 18-years of age, with human immunodeficiency virus, hepatitis B or hepatitis C virus infection, pregnancy, liver transplantation, supplements and foods with probiotics, immunosuppressants, alcohol intake (greater than 15 g ethanol/day), antibiotic use in the last 6-months, and any other chronic inflammatory diseases were excluded. The control group consist of healthy individuals who did not have chronic or viral diseases, were not taking prescribed medication and had not used antibiotics in the last 6-months.

This study was conducted in accordance with the Declaration of Helsinki. All procedures involving human subjects were approved by the ethics and research committee of the Hospital de Clínicas de Porto Alegre (Approval n° 94281118.7.0000.5327). The Written informed consent was obtained from all subjects. The methodology used in this study followed the STARD guidelines.^17^

### Clinical design

Our cohort consisted of 5 Healthy Control (CN), 6 steatohepatitis (MASH), 7 Cirrhosis (CIR) and 5 Hepatocellular Carcinoma (HCC) patients. Samples were collected between 2022 and 2023, from patients of both sexes who met the inclusion criteria. Stool samples were collected in sterile packaging and kept refrigerated until arrival at the laboratory (HCPA gastroenterology and hepatology laboratory), then were stored in a 80 °C freezer until subsequent analyses. All clinical, biochemical (Aspartate Aminotransferase [AST] Alanine Aminotransferase [ALT], Gamma-Glutamyltransferase [GGT], Alkaline Phosphatase [AP], bilirrubin, albumin, creatinin, platelets, glucose, HbA1c, cholesterol, triglycerides) and demographic data (ethnicity, gender, age, BMI, smoking, alcohol use, medicines use, degree of fibrosis) were collected retrospectively from the patients' medical records. The MASLD diagnosis was made by biopsy (MASH group) or by consulting the medical records of patients` medical records and reviewing laboratory, image and endoscopic exams (HCC and cirrhosis groups).

### DNA extraction and 18S rRNA sequencing

Fungal DNA from stool samples was extracted using the *Nova Biotecnologia* Soil DNA Kit (cat. 13-BR700S-50, Nova Biotecnologia, Brazil), following the manufacturer's instructions. This protocol includes a bead-beating step with ceramic microbeads combined with a chaotropic and detergent-based lysis buffer, ensuring efficient disruption of fungal cell walls composed of chitin and β-glucans and allowing unbiased recovery of fungal DNA from complex matrices. DNA integrity was assessed by agarose gel electrophoresis, and concentrations were measured fluorometrically using a Qubit™ 2.0 (Life Technologies, USA).

### PCR amplification, library preparation, and sequencing (18S V9)

The V9 region of the 18S rRNA gene was amplified using primers 1380F (5′-CCCTGCCHTTTGTACACAC-3′) and 1510R (5′-CCTTCYGCAGGTTCACCTAC-3′) (Amaral-Zettler et al., 2009) with Phusion High-Fidelity Master Mix (New England Biolabs). Each extraction batch included a blank extraction control, and each PCR plate included a no-template control. These controls consistently yielded no visible amplicons and produced no sequencing reads above background. Only samples that successfully amplified proceeded to library preparation and sequencing. Amplicons (∼400–450 bp including adapters) were verified on 2% agarose gels, purified with AMPure XP beads, and quantified with a Qubit fluorometer. Libraries were prepared through single-step indexing, in which Illumina adapter tails and dual indexes were incorporated during amplification, followed by adapter ligation. Indexed amplicons were normalized, pooled equimolarly, and sequenced on an Illumina NovaSeq 6000 platform using paired-end 2 × 250 bp reads with a 1%–5% PhiX spike-in. Clean FASTQ files (≥ 40,000 paired reads per sample) were generated after standard Illumina quality filtering.

The 18S rRNA V9 region was selected for amplification because ITS1 and ITS2 primers did not yield consistent results across samples, likely due to low fungal biomass and host DNA interference. Taxonomic assignment was performed using the SILVA v138 database, which provides curated 18S rRNA coverage for both fungal and eukaryotic taxa, whereas UNITE is primarily curated for ITS regions.

### Scientific justification for the 18S-V9 marker and primers

The 18S rRNA V9 region was chosen as a universal eukaryotic barcode capable of detecting a broad spectrum of intestinal fungi while maintaining short amplicon size suitable for degraded or low-biomass DNA typical of stool samples.^18^ Perlin et al. (2023) highlighted that, among liver- and gut-related mycobiome studies, both ITS and 18S rRNA regions are used, but 18S provides higher amplification consistency and inter-study comparability across diverse eukaryotic taxa.^14^ Borges et al. further confirmed that 18S rDNA sequencing reliably identified and validated fungal isolates from human feces, demonstrating its robustness for profiling gut mycobiota.^19^ Following these findings, our study adopted the primers 1380F/1510R, which target conserved regions flanking V9 and are recommended in current QIIME2/SILVA 138 workflows. This choice enables genus-level resolution, facilitates integration with global eukaryotic datasets, and minimizes amplification bias observed in ITS-based analyses of human fecal DNA.

### Mycobiota analysis and statistics

Amplicon Sequence Variants (ASVs) were inferred using the DADA2 pipeline (filtering, trimming, denoising, merging of paired reads, and ASV table construction).^20^ Taxonomy was assigned with the naïve Bayesian classifier against the SILVA v138 reference database.^21^ To focus on the mycobiota, only ASVs assigned to the phyla Ascomycota and Basidiomycota were retained for downstream analyses. Visualization and diversity analyses were conducted in R (R Core Team, 2019) using phyloseq, vegan, and ggplot2.^22^, ^23^, ^24^, ^25^ Alpha diversity differences between groups were evaluated with the Wilcoxon rank-sum test. Beta diversity was assessed by NMDS on Bray-Curtis dissimilarities, and group separation was tested by PERMANOVA. To identify differentially abundant taxa, we used DESeq2 with the Wald test.^26^ Multiple testing was controlled using the Benjamini-Hochberg procedure, and significance was determined by adjusted p-values (padj) with a threshold of padj < 0.05 (unless otherwise stated). In addition, candidate differentially abundant ASVs were manually curated to mitigate spurious findings: features driven by extreme abundance in a single sample and not representative of group-level patterns were excluded from reporting.

## Results

### Study population

A total of 28 participants were initially recruited (6 controls, 8 MASH, 8 cirrhosis, and 6 HCC). However, due to DNA extraction losses, 23 samples were successfully sequenced and included in the final analysis. The study included 23 participants: MASH (n = 6), CIR (n = 7), HCC (n = 5), and CN (n = 5). Patients with MASLD-spectrum conditions were older and had higher BMI compared with CN (Table 1). Liver disease markers were higher in MASLD groups than in CN. Most participants were non-smokers 91.3% (21/23) and reported no current alcohol use 82.6% (19/23). Metabolic syndrome was present in 66.7% of MASH (4/6) and in 100% of CIR (7/7) and HCC (5/5), totaling 88.9% (16/18) among MASLD patients. Regarding metabolic parameters (Table 1), MASLD groups showed higher fasting glucose, HbA1c, triglycerides, and total cholesterol than CN group. Chronic medication use was reported by 56.5% (13/23), including antihypertensives and/or statins*.*

**Table 1:** Sociodemographic and clinical characteristics of the population.

	^a^Control (n = 5)	^b^MASH (n = 6)	^c^Cirrhosis (n = 7)	^d^HCC (n = 5)	p-value
**Gender [female], n (%)**	5 (100%)	3 (50%)	7 (100%)	2 (40%)	>0.05
**Ethnic [White] n (%)**	4 (80%)	5 (83.3%)	5 (71.4%)	5 (100%)	>0.05
**Age [years]**	31.2 ± 3.2	58.2 ± 11.9	54.6 ± 20.8	75 ± 2.4	0.001**^a,b^** | 0.049**^a.c^**| 0.001**^a.d^**
**BMI [kg/m^2^]**	27.3 ± 5.1	32.7 ± 4.4	32.2 ± 5.8	28.2 ± 4.1	>0.05
**ALT [IU/L]**	31 ± 1.5	56.2 ± 20.2	36.6 ± 22.8	43.4 ± 17.7	0.03**^a,b^**
**AST [IU/L]**	32.2 ± 1.9	47.8 ± 20	44.2 ± 15.1	43.4 ± 20.1	>0.05
**GGT [IU/L]**	25.4 ± 1.1	47.8 ± 20	304.2 ± 336	222 ± 137	0.01**^a,c^** | 0.03**^a.d^**
**AP [IU/L]**	77.4 ± 7.9	101.8 ± 34.3	195.8 ± 147.7	147.4 ± 64.3	>0.05
**Bilirubin [mg/dL]**	0.48 ± 0.31	0.44 ± 0.16	0.98 ± 0.38	1.62 ± 1.55	>0.05
**Albumin [g/dL]**	4.02 ± 0.39	4.31 ± 0.35	3.9 ± 0.93	3.98 ± 0.58	>0.05
**INR**	1 ± 0	1 ± 0	1.12 ± 0.13	1.17 ± 0.33	0.01**^a.c^**
**Creatinine [mg/dL]**	0.74 ± 0.16	0.90 ± 0.18	1.33 ± 1.01	1.33 ± 1.01	>0.05
**Platelets [10^9^/L]**	250 ± 79.05	285 ± 78.7	119.8 ± 0.16	239.6 ± 178	0.02 **^a.c^**
**Glucose [mg/dL]**	86.6 ± 9.63	250 ± 79.05	142.4 ± 44.3	181.2 ± 116.8	0.04**^a,b^** | 0.01**^a.c^**
**HbA1c [%]**	3.92 ± 0.7	6.34 ± 1.1	7.24 ± 3.06	6.58 ± 1.64	0.01**^a,b^** | 0.01**^a.c^**| 0.02**^a.d^**
**Triglycerides [mg/dL]**	73 ± 12.04	95.8 ± 26.9	126.2 ± 62.6	136.4 ± 48.7	0.02 **^a.c^** | 0.04**^a.d^**
**Total cholesterol [mg/dL]**	114 ± 20.7	172.4 ± 46.2	174 ± 44.6	158.6 ± 22.2	0.01 **^a.c^**| 0.04**^a.d^**
**HDL cholesterol [mg/dL]**	40.8 ± 5.6	42 ± 19.1	35.2 ± 10	38.6 ± 10.2	>0.05
**MetS, n (%)**	0 (0%)	4 (66.66%)	7 (100%)	5 (100%)	0.001**^a,c^** | 0.008**^a.d^**
**Smoking, n (%)**	0 (0%)	2 (33.33%)	0 (0%)	0 (0%)	>0.05
**Alcohol use n (%)**	0 (0%)	3 (50%)	0 (0%)	1 (25%)	>0.05
**Medicines use n (%)**	0 (0%)	3 (50%)	6 (85,7%)	4 (80%)	0.015**^a,c^** | 0.047**^a.d^**

Values are mean ± SD for continuous variables and n (%) for categorical variables. The global p-value compares the four groups (one-way ANOVA or Kruskal-Wallis, as appropriate). Superscript letters denote groups: a = Control, b = MASH, c = Cirrhosis, d = HCC. Superscript letter-pairs next to a p-value indicate significant post-hoc pairwise comparisons after correction for multiple testing (^a,b = Control vs. MASH; ^a,c = Control vs. Cirrhosis; ^a,d = Control vs. HCC). The symbol “>” indicates a non-significant result (p > 0.05). Units are mg/dL unless otherwise specified.

ALP, Alkaline Phosphatase; ALT, Alanine Aminotransferase; AST, Aspartate Aminotransferase; BMI, Bdy Mass Index; GGT, Gamma-Glutamyltransferase; HbA1c, Glycated Hemoglobin A1c; HDL, High-Density Lipoprotein cholesterol; INR, International Normalized Ratio; MetS, Metabolic Syndrome; Units, 10^9/L, Billions per liter; IU/L, International Units per liter; mg/dL, milligrams per deciliter.

### Fecal mycobiome changes composition in MASLD patients and controls

Beta diversity did not separate MASLD from controls (Bray-Curtis PERMANOVA visualized by NMDS; p p = 0.571, Fig. 1). In contrast, alpha diversity differed across groups by Simpson’s index and was higher in MASLD than in controls (MASH = 0.879, CIR = 0.782, HCC = 0.766 vs. CN = 0.425; Fig. 2), with MASH vs. CN (p = 0.03576) and MASH vs. CIR (p = 0.01842) reaching significance (Wilcoxon).

**Fig. 1:**
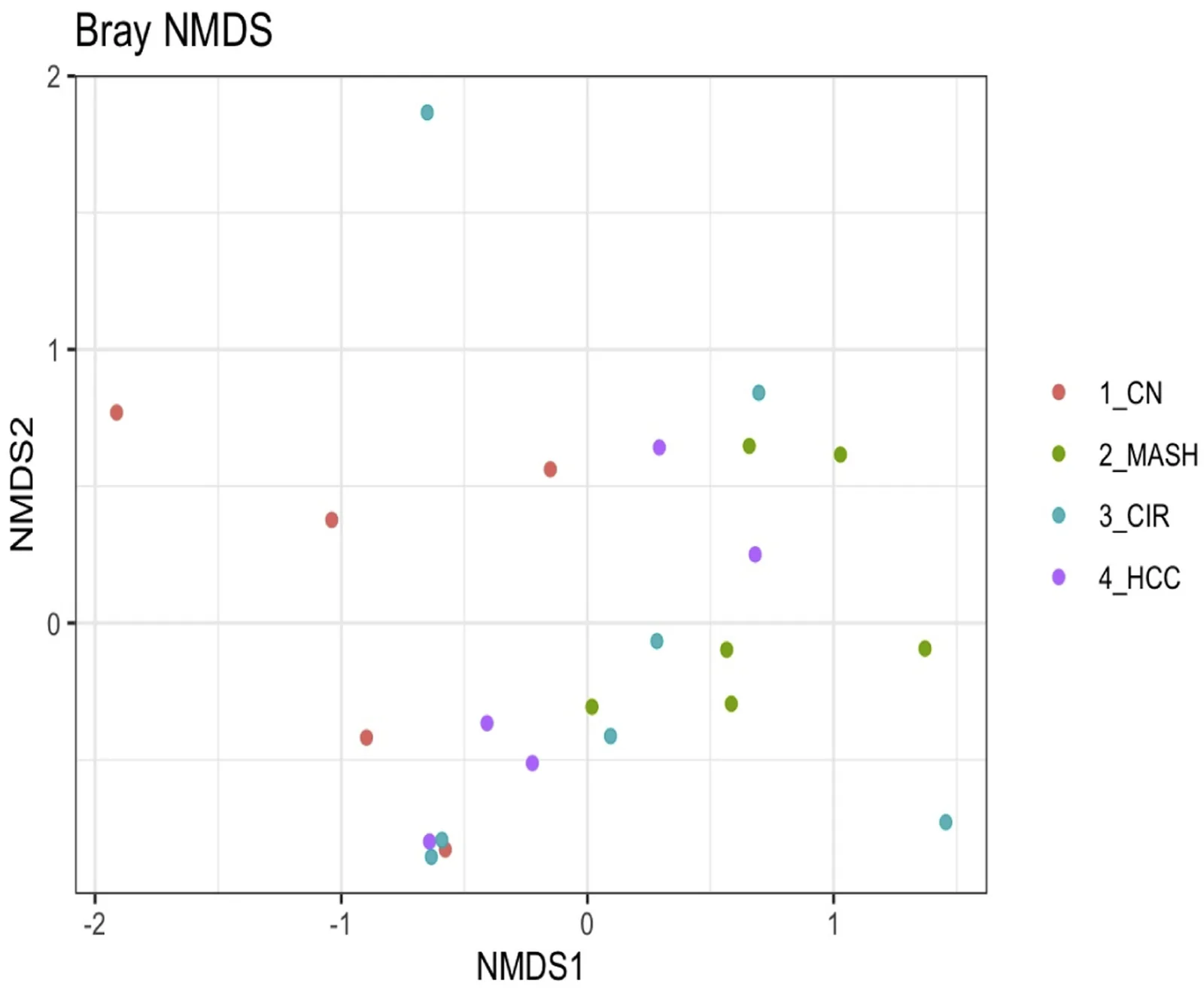
Beta diversity analyses of the fecal mycobiome. Bray-Curtis dissimilarities visualized by NMDS; groups colored (CN, orange; MASH, green; CIR, blue; HCC, lilac). Group differences assessed with PERMANOVA.

**Fig. 2:**
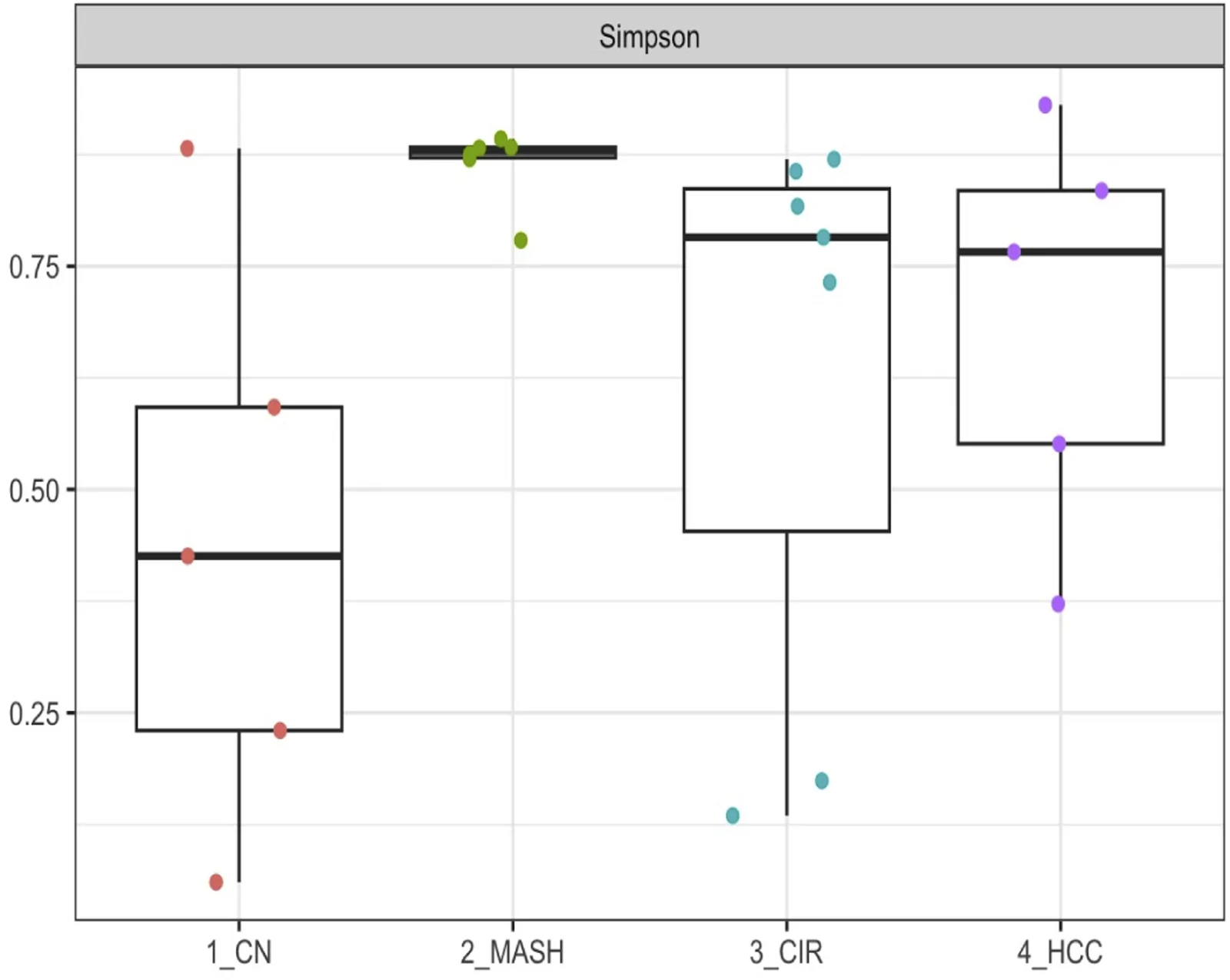
Alpha diversity analyses of the fecal mycobiome. (Simpson’s index) by group (CN, orange; MASH, green; CIR, blue; HCC, lilac). Boxplots show median and IQR with individual samples overlaid. Pairwise comparisons assessed with Wilcoxon rank-sum tests.

At the phylum level, Ascomycota predominated in CN, whereas Basidiomycota was relatively higher in MASH (Fig. 3). At the family level, *Aspergillaceae* was more abundant in CN; *Malasseziaceae, Filobasidiaceae* and *Sporidiobolaceae* in MASH; Malasseziaceae and *Saccharomycetaceae* in CIR; and *Saccharomycetaceae* in HCC (Fig. 4). At the genus level, CN showed higher relative abundance of *Penicillium*; MASH showed higher *Malassezia* and *Naganishia* (with a larger fraction of unclassified genera); CIR was enriched for *Saccharomyces*; and HCC showed higher *Malassezia* and *Saccharomyces* (Fig. 5).

**Fig. 3:**
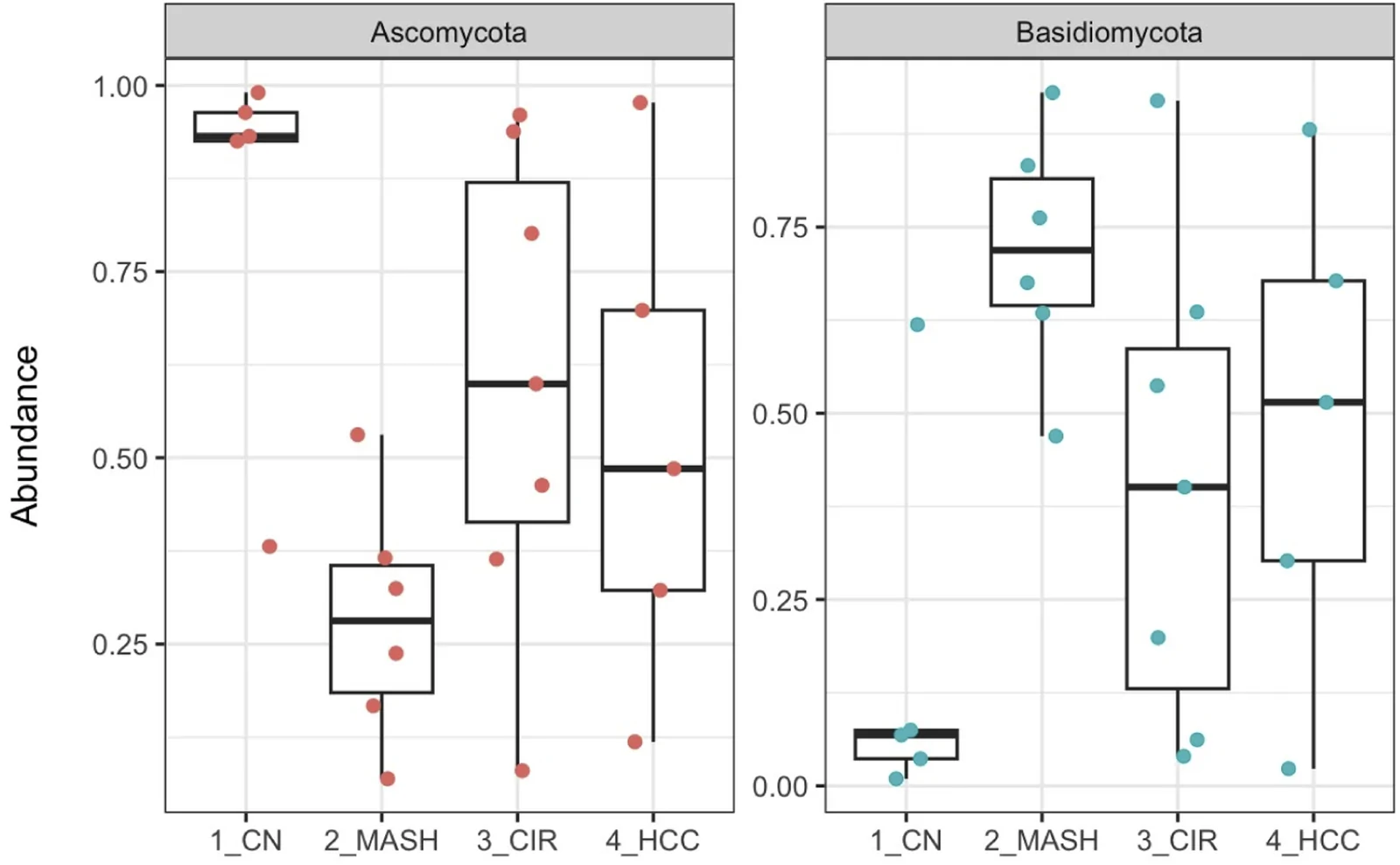
Phylum-level composition (relative abundance per sample; stacked bars). The phylum-level taxonomic profile of mycobiome.

**Fig. 4:**
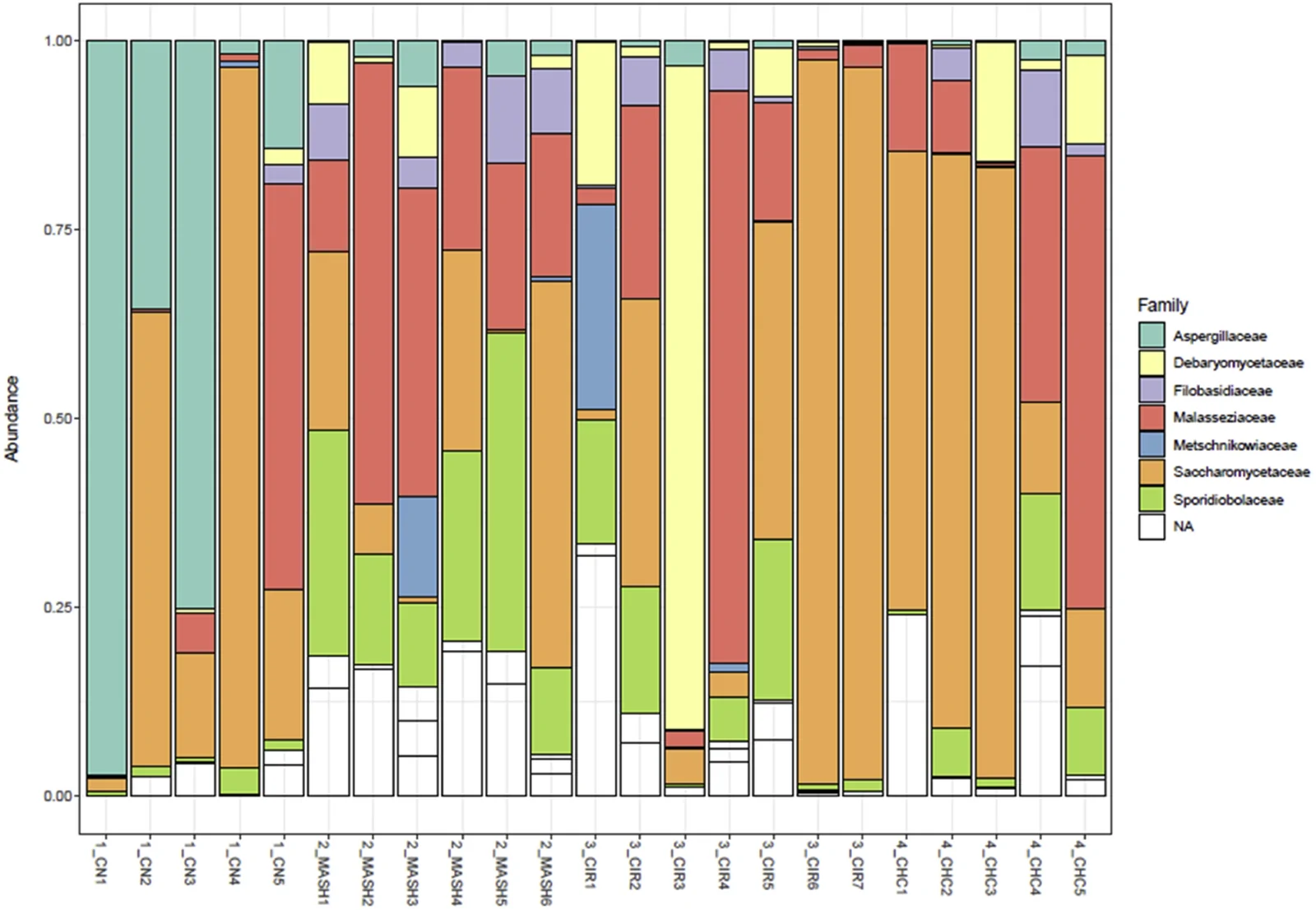
Family-level composition (relative abundance per sample; stacked bars). “NA” denotes unclassified reads. The family-level taxonomic profile of mycobiome.

**Fig. 5:**
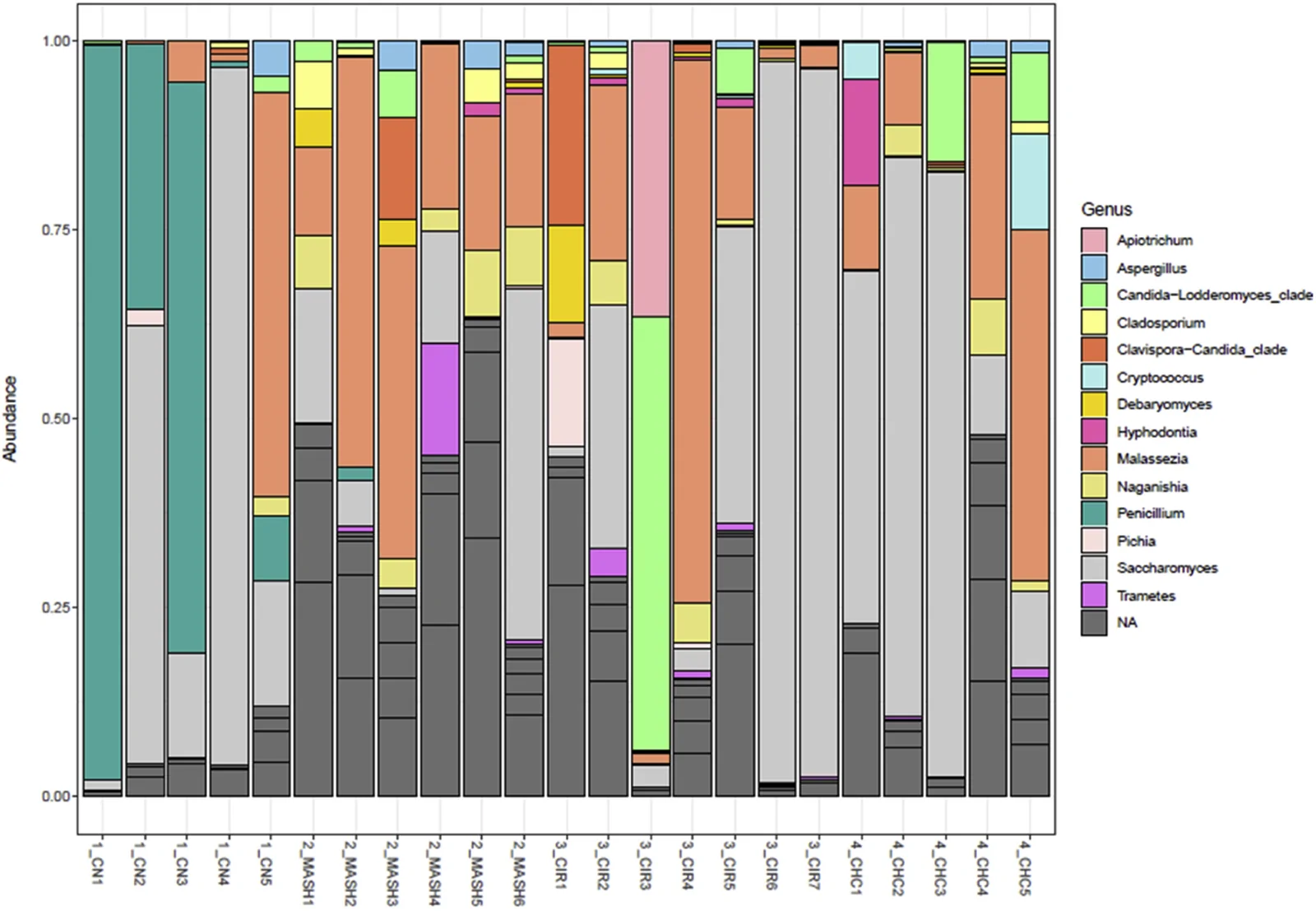
Genus-level composition (relative abundance per sample; stacked bars). “NA” denotes unclassified reads. The genus-level taxonomic profile of mycobiome.

Differential abundance analysis with DESeq2 identified three ASVs differing between CN and MASLD (ASV20, ASV65, ASV8; Fig. 6A). When stratified by group, ASV20 and ASV8 were more abundant in MASH, whereas ASV65 was more abundant in HCC (Fig. 6B).

**Fig. 6:**
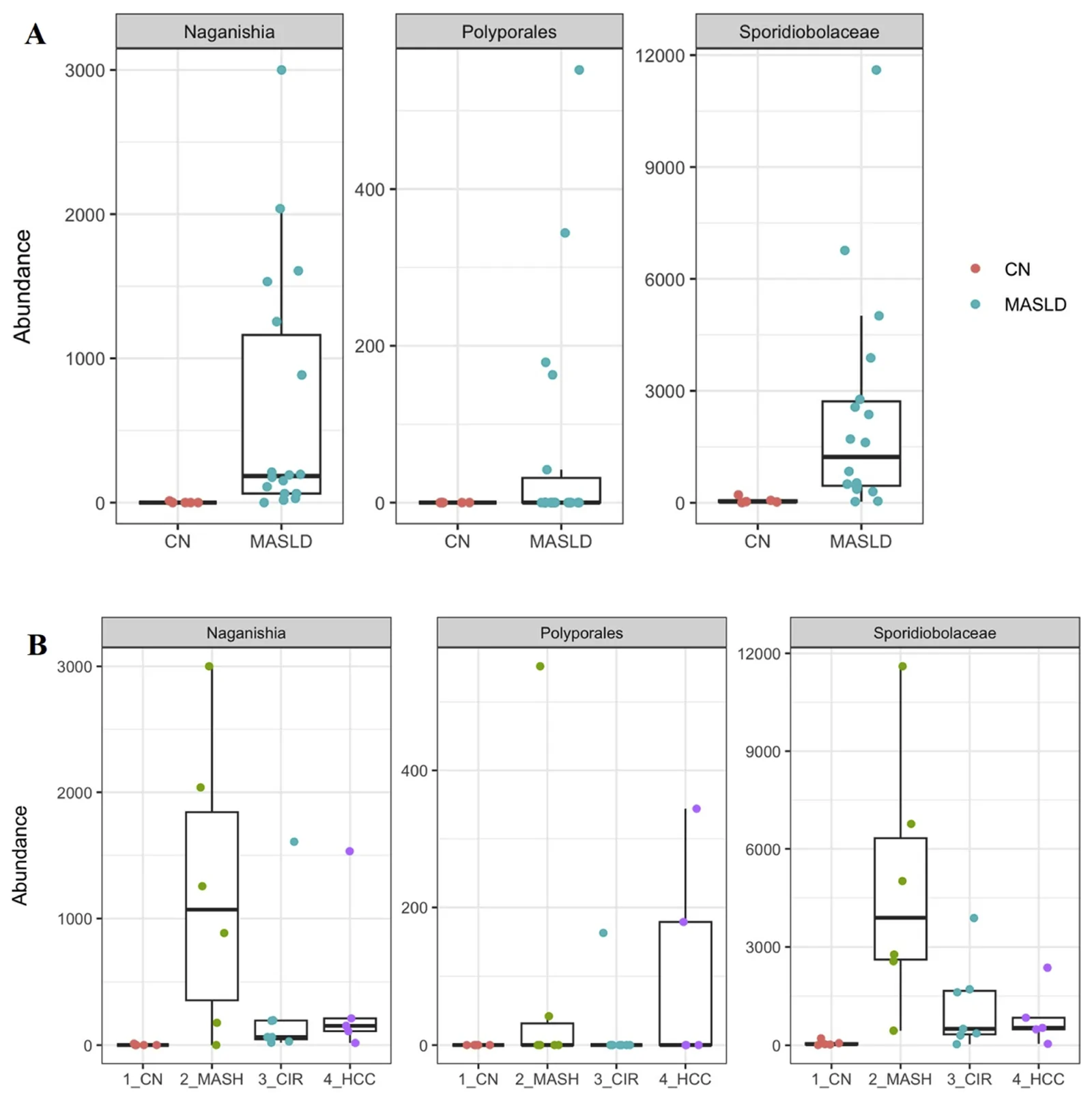
Differential abundance of selected ASVs analyzed by DESeq2 (normalized counts). (A) Comparison between Controls (CN) and MASLD group. (B) Stratified by group (CN, MASH, CIR, HCC). Statistical testing performed with DESeq2’s negative binomial model. Mycobiome differential abundance analysis.

## Discussion

Our study provides one of the first fungal characterizations across the full MASLD spectrum within a single human cohort, showing that the abundance of specific taxa varies across disease stages. While overall fungal community structure remained broadly similar between groups, certain taxa were enriched in specific stages: Ascomycota/*Penicillium* in controls, Malassezia and *Naganishia* in MASH, and Saccharomyces in cirrhosis/HCC. Notably, the presence of *Naganishia* in MASH has not been previously reported in MASLD patients, though it has been observed in other metabolic or inflammatory conditions. These findings suggest that shifts in particular fungal taxa accompany disease progression, even in the absence of major changes in global beta diversity.

In our cohort, beta diversity did not distinguish the groups based on overall fungal composition, whereas alpha diversity did show significant differences, with higher values in MASLD compared to controls, reaching statistical significance in MASH. This partially aligns with MASLD/NAFLD studies where beta-diversity analyses found no differences in overall comparisons (controls vs. NAFLD strata) but observed significant separation after stratifying by obesity, restricted to non-obese patients (e.g., NASH vs. NAFL; F2–F4 vs. F0–F1), persisting after adjustment for age, sex, alcohol, BMI, PPI use, and T2D.^27^ In contrast, other MASLD cohorts have reported beta-diversity differences between patients and controls with ITS-based pipelines, or between disease groups (e.g., MAFLD vs. ALD) using metrics such as Jaccard/Bray-Curtis.^28^^,^^29^ Findings for alpha diversity similarly diverge across studies and methods: some report no alpha-diversity differences (Shannon, inverse Simpson) across MAFLD/ALD/controls, whereas others report higher alpha (observed species and Shannon) in NAFLD vs. controls and in NASH vs. non-NASH.^27^, ^28^, ^29^ In advanced disease, beta-diversity clustering has been observed for HCC vs cirrhosis and controls, with lower alpha diversity in HCC.^30^ Overall, the variability in alpha and beta diversity signals across studies seems to depend on cohort structure (e.g., obesity strata, disease stage), methodological choices (marker, database, distance/alpha metrics), and adjustment for confounders (age, sex, BMI, medication), which helps explains our finding of no global beta-diversity separation alongside differences in alpha diversity between groups. These considerations provide context for interpreting our cohort's fungal composition and the potential relevance of specific taxa in MASLD progression.

Several of the taxa identified in our cohort have been implicated in hepatic and gastrointestinal diseases. Malassezia has been associated with inflammation and fibrosis mediated by complement activation in murine models, whereas Saccharomyces and Candida have been linked to dysbiosis and gastrointestinal cancer. In our study, their enrichment in advanced MASLD stages may reflect inflammatory or metabolic conditions characteristic of advanced disease. These observations are consistent with previous studies, although they should be considered strictly descriptive and not as functional evidence.^29^^,^^30^

Fungal profiles in stool reflect a mixture of transient dietary/environmental inputs and taxa selected by gut niche conditions. Along the MASLD spectrum, shifts in luminal lipids, bile acids, low-grade inflammation, mucus, and barrier permeability can reshape which fungal guilds thrive ‒ often favoring lipid-dependent basidiomycetes in metabolically altered states and fermentative ascomycetes in advanced disease.^29^, ^30^, ^31^, ^32^, ^33^ At the phylum level, we observed Ascomycota predominated in controls ‒ consistent with a larger contribution of dietary and environmental molds and yeasts in healthy stools ‒ whereas Basidiomycota showed relatively higher abundance in MASH, in keeping with lipid-rich and bile-altered niches that can support basidiomycetous yeasts.^31^, ^32^, ^33^ At the family level, Aspergillaceae in controls aligns with transient dietary/ambient exposure, while *Malasseziaceae, Filobasidiaceae*, and *Sporidiobolaceae* in MASH are compatible with Basidiomycete-leaning communities under metabolic stress. In cirrhosis/HCC, the prominence of *Saccharomycetaceae* fits scenarios of barrier impairment and readily fermentable substrates in advanced disease.^29^^,^^30^^,^^33^ At the genus level, *Penicillium* in controls reflects healthy/eutrophic stools, *Malassezia* in MASH matches its lipid-dependent ecology and recurrent appearance in metabolic-hepatic contexts, and *Saccharomyces* in cirrhosis/HCC aligns with reports in advanced liver disease and diet-induced models.^29^, ^30^, ^31^, ^32^, ^33^, ^34^, ^35^ We additionally observed an association of *Naganishia* with MASH; in other metabolic disease settings, *Naganishia* has been reported as reduced in type-2 diabetes compared with healthy controls, though findings are variable across cohorts and methods.^34^ These contrasting signals suggest that *Naganishia* may mark subgroup-specific niches within the MASLD spectrum and merits confirmation in larger, matched cohorts with standardized profiling.

Environmental and lifestyle factors may also shape these mycobiome profiles. Our cohort, recruited in southern Brazil, is characterized by high consumption of yerba mate (*Ilex paraguariensis*) and red meat ‒ two elements with reported microbiota-modulating effects.^36^^,^^37^ Such regional habits could partially explain differences compared to Asian or European MASLD cohorts. Technical and analytical constraints should be acknowledged. The complex fungal cell wall complicates DNA extraction, and many fungal species remain uncultivable.^38^^,^^39^ Methodological variation across studies (ITS region, database, sequencing depth) further limits direct comparisons. Our sample size was modest, underscoring the need for validation in larger, matched cohorts.

Separately, our data show that ASVs were concordant with the taxonomic profiles: features mapping to basidiomycetous lineages in MASH and a feature aligned with *Saccharomycetaceae* in HCC, reinforcing the stage-wise pattern. Because 18S V9 tends to resolve higher taxonomic ranks and assignments depend on reference coverage/curation (e.g., UNITE vs. SILVA), a larger share of reads can remain “unclassified” ‒ as we observed ‒ whereas ITS1/ITS2 generally improves genus/species-level resolution; this has been emphasized in recent reviews and healthy-reference cohorts.^32^^,^^40^ Taken together, these feature-level signals provide a short list for targeted confirmation using higher-resolution profiling (e.g., ITS/UNITE) or qPCR, and for subsequent testing of clinical associations.

In summary, our study provides a preliminary characterization of the fecal mycobiome across the MASLD spectrum, highlighting stage-specific shifts in taxa such *as Penicillium, Malassezia, Naganishia, and Saccharomyces*. These observations suggest that fungal community composition varies with disease progression, although overall beta diversity remained largely unchanged. However, these findings must be interpreted cautiously, considering the small and demographically unmatched groups, the use of 18S V9 rather than ITS regions (after failed ITS1/ITS2 amplification) and the consequent reliance on SILVA v138 instead of UNITE, and potential methodological biases in DNA extraction and taxonomic assignment. Future studies with larger cohorts, matched for age and BMI, using higher-resolution fungal profiling and rigorous statistical controls, are needed to validate these stage-specific fungal signatures and explore their potential mechanistic or clinical relevance in MASLD.

## Statement of ethics

The study was approved by the Research Ethics Committee – Hospital de Clínicas de Porto Alegre. It was conducted in accordance with local legislation and institutional requirements. Participants provided their written informed consent to participate in this study.

## Declaration of generative AI

During the preparation of this work, the authors used ChatGPT for grammar correction and text refinement. Authors take full responsibility for the integrity of the content generated.

## Authors’ contributions

Perlin CM, Keingeski MB, Basso BS and Álvares-da-Silva MR performed the conceptualization, methodology, formal analysis, investigation, data curation, writing of the original draft, writing-review, and editing; Mendes BG and Schnabl B, performed consulting and advising on methodology and design, and revised the manuscript, Medeiros JD and Borges FM, performed the methodology and formal analysis. All authors participated in the final revision and approval of the manuscript.

## Funding

This study was financed in part by the National Council for Scientific and Technological Development, CNPq (MRAS), FIPE Hospital de Clínicas de Porto Alegre (ROR: https://ror.org/010we4y38) (MRAS), and Coordenação de Aperfeiçoamento de Pessoal de Nível Superior – Brasil (CAPES) – Finance Code 00 (CMP, MBK). BS is supported by NIH grant P30 DK120515.

## Data availability

Raw FASTQ files will be deposited in the NCBI SRA repository upon authorization from the data owner. Accession numbers will be provided once available.

## Conflicts of interest

The authors declare no conflicts of interest.
